# Evaluation of Adherence to Oral Hypoglycemic Agent Prescription in Patients with Type 2 Diabetes

**DOI:** 10.1900/RDS.2020.16.41

**Published:** 2021-05-01

**Authors:** Mahtab Irani, Mohammad Sarafraz Yazdi, Meisam Irani, Sina Naghibi Sistani, Sahar Ghareh

**Affiliations:** 1 Faculty of Medicine, Islamic Azad University, Mashhad branch, Mashhad, Iran.; 2 Department of Internal Medicine, Mashhad Medical Sciences Branch, Islamic Azad University, Mashhad, Iran.; 3 Faculty of Medicine, Shahrood Medical Sciences Branch, Islamic Azad University, Shahrood, Iran.; 4 Department of Psychology, Ferdowsi University of Mashhad, Faculty of Education Sciences and Psychology, Mashhad, Iran.; 5 Department of Endocrinology, Mashhad Medical Sciences Branch, Islamic Azad University, Mashhad, Iran.

**Keywords:** medication adherence, hypoglycemic drugs, type 2 diabetes

## Abstract

**BACKGROUND:**

Diabetes is a global health problem that has affected more than 400 million people worldwide. Adherence to treatment is considered to be one of the most impor tant and deterministic factors in the treatment of diabetes. This study investigates medication adherence and factors af fecting it in patients with type 2 diabetes.

**METHODS:**

This cross-sectional study investigated 136 patients with type 2 diabetes in 2018-2019. Data collection was done using a checklist that included information on personal characteristics, medication, and healthcare. The collected data were analyzed by statistical tests in SPSS 25 software.

**RESULTS:**

79.4% of the patients adhered to prescribed medication. Medication adherence had no significant relationship with taking other drugs, fasting blood sugar (FBS), and the daily number of hypoglycemic tablets (p ˃ 0.05). However, adherence to medication was significantly associated with age, gender, income, hemoglobin A1c, medication period, and hypoglycemia (p < 0.05).

**CONCLUSIONS:**

Higher levels of adherence were` obser ved among females aged below 60 years, with higher income, a hemoglobin A1c level below 7%, a medication period of less than 10 years, and among patients without hypoglycemia. Regarding drug type, adherence levels were lower in people taking glibenclamide.

## 1 Introduction

Diabetes is one of the most critical epidemic diseases all over the world [1]. Type 2 diabetes (T2D) is highly prevalent, and is considered a metabolic chronic disease with consequences affecting public health and healthcare cost [2]. In 2017, more than 400 million people were diagnosed with T2D worldwide [3]. Diabetes can be controlled by a healthy lifestyle and on-time medication. Uncontrolled diabetes may cause microvascular and macrovascular complications [4]. Vascular complications may be prevented by controlling blood glucose levels in patients with T2D [5, 6]. Increased prevalence rates of diabetes impose individual, familial, and health-related challenges on people and the global systems, and are accompanied by increased mortality and morbidity [7]. Beside using hypoglycemic drugs, changes in lifestyle and diet may help to improve glycemic control and to achieve target blood glucose levels in patients before starting insulin therapy [8]. Effective treatment of T2D comprises an integrated treatment of lifestyle modification and medication intervention. Important factors in achieving optimal glycemic control are treatment by hypoglycemic drugs and medication adherence [9, 10].

Medication adherence is decisive in controlling T2D [11]. Various factors determine medication adherence of diabetic patients in their daily life, including degree of disease complications, kind of treatment, age, gender, existence of stress and depression, and number of drugs included in the therapy [12]. Poor adherence is accompanied by the risk of hospitalization and the development of diabetes complications such as kidney diseases [13]. If used properly, orally ingested hypoglycemic drugs are highly effective in controlling T2D [14]. In general, high rates of adherence to hypoglycemic drugs lead to lower numbers of drugs being used [15].

T2D is usually diagnosed with a high level of hemoglobin A1c, and drug therapy is applied based on hypoglycemic effects, patients’ priorities, and drug side effects [16]. Oral treatment with metformin, sulphonylurea, thiazolidinediones (TZD), and dipeptidyl peptidase-4 inhibitor (DPP4) does not result in definite cure, but rather improved glycemic control, prevention of disease complications, and mitigation of symptoms [17].

T2D causes a wide range of microvascular and macrovascular complications, including cardiovascular, kidney, and eye diseases, blindness, neuropathy, amputation, and also nonvascular complications; all these complications impose a lot of pain and discomfort on patients [18]. Adherence to hypoglycemic drugs has been proven to be an effective and cost-efficient means to reduce diabetes complications and hospitalization times and thus to reduce the cost of short- and long-term complications [19]. Since various studies have investigated patients’ adherence to oral hypoglycemic drugs, we aimed to investigate the factors involved in adherence to or noncompliance with medication prescriptions and the ratio of the number of days for which the patient takes the drug to the number of days in 6 months.

## 2 Methods

### 2.1 Study design

This cross-sectional study was performed to investigate medication adherence among 136 patients with T2D between September 2018 and March 2019. The participants were selected from patients referred to the clinics of internal diseases and endocrinology, which are affiliated with Islamic Azad University, and they gave informed consent. The samples were selected by convenience sampling, and data collection was done using a checklist designed to evaluate the data on gender, age, drug affordability, hypoglycemia, type of drug, number of daily tablets used for diabetes, medication period, concomitant drugs, fasting blood glucose, and hemoglobin A1c. The follow-up investigation was performed after 6 months, and finally, the ratio of the number of days for which the patient had taken the drug to the number of follow-up days was calculated. In the end, all collected data were analyzed by SPSS 25 software.

### 2.2 Inclusion and exclusion criteria

Patients were included if they fulfilled the following two criteria:

- Having type 2 diabetes
- Using oral hypoglycemic drugs

Patients were excluded if one or more of the following criteria applied:

- Unwillingness to participate in the study
- Taking insulin
- Any change in the type of diabetes drugs taken over the past 6 months

### 2.3 Data analysis

The data analysis was done by using SPSS 25 software at the significance level of less than 5%. Values below 5% are indicated by “*”, and values below 1% are indicated by “**” in the tables.

### 2.4 Ethical considerations

The following list of ethical considerations was applied:

- No extra cost was imposed on patients in this study.
- The patients’ information was and will be kept private.
- The patients participated in the study if they were willing to do so.

## 3 Results

The present study investigated the adherence to hypoglycemic drugs and its association with gender, age, income, hemoglobin A1c, fasting blood glucose (FBG), medication period, hypoglycemia, concomitant drugs, daily number of hypoglycemic tablets used, and type of drug.

Table 1 indicates that there were no significant associations between medication adherence and using concomitant drugs, FBG, and the daily number of hypoglycemic tablets used. However, there were significant relationships between medication adherence and age, gender, income, hemoglobin A1c, medication period, and hypoglycemia (p < 0.05). Of the 136 diabetes patients, 85 (62.5%) were women and 51 (37.5%) were men. Adherence to oral hypoglycemic drugs was reported for 108 patients (79.4%), and 28 patients (20.6%) reported noncompliance with medication. Table 2 presents medication adherence in terms of the type of drug. According to the data, medication adherence was significantly affected in patients who took glibenclamide (p = 0.048), i.e. medication adherence was lower in patients taking glibenclamide.

**Table 1.** Medication adherence in patients with type 2 diabetes

Variable		Adherence		Noncompliance		Total	
		Number	Percentage (%)	Number	Percentage (%)	Statistic	p
Gender	Male	73	85.9	12	14.1	5.64	00.018*
	Female	35	68.6	16	31.4		
Age	< 60 years old	58	86.6	9	13.4	4.21	0.040*
	≥ 60 years old	50	72.5	19	27.5		
Income	< 3 million Tomans	57	75.0	19	25.0	8.24	0.016*
	2-4 million Tomans	35	79.5	9	20.5		
	≤ 4 million Tomans	16	100.0	0	0.0		
Hemoglobin A1c	< 7	64	87.7	9	12.3	6.64	0.010*
	≤ 7	44	69.8	19	30.2		
Blood sugar	< 130	60	84.5	11	15.5	2.36	0.124
	≤ 130	48	73.8	17	26.2		
Hypoglycemia	Positive	42	65.6	22	34.4	14.62	0.0001**
	Negative	66	91.7	6	8.3		
Medication period	< 10	73	84.9	13	15.1	4.16	0.041*
	≤ 10	35	70.0	15	30.0		
Number of hypoglycemic tablets	1	13	92.9	1	7.1	1.92	0.430
	2-5	87	77.0	26	23.0		
	< 5	8	88.9	1	11.1		
Other drugs	Yes	99	78.6	27	21.4	0.86	0.353
	No	9	90.0	1	10.0		

**Table 2.** Medication adherence by the type of drug used by type 2 diabetic patients

Medicine		Adherence		Noncompliance		Total	
		Number	Percentage	Number	Percentage	Number	Percentage
Metformin	Using	86	77.5	25	22.5	111	100.0
	Not using	22	88.0	3	12.0	25	100.0
	Test statistic, p-value	Likelihood ratio = 1.53 p-value = 0.287					
Glibenclamide	Using	43	71.7	17	28.3	60	100.0
	Not using	65	85.5	11	14.5	76	100.0
	Test statistic, p-value	Likelihood ratio = 3.92 p-value = 0.048*					
Gliclazide	Using	21	84.0	4	16.0	25	100.0
	Not using	87	78.4	24	21.6	111	100.0
	Test statistic, p-value	Likelihood ratio = 0.41 p-value = 0.512					
Linagliptin	Using	3	100.0	0	0.0	3	100.0
	Not using	105	78.9	28	21.1	133	100.0
	Test statistic, p-value	Fisher’s exact test p-value = 0.999					
Repaglinide	Using	5	71.4	2	28.6	7	100.0
	Not using	103	79.8	26	20.2	129	100.0
	Test statistic, p-value	Fisher’s exact test p-value = 0.633					
Sitagliptin	Using	11	73.3	4	26.7	15	100.0
	Not using	97	80.2	24	19.8	121	100.0
	Test statistic, p-value	Fisher’s exact test p-value = 0.511					
Pioglitazone	Using	4	100.0	0	0.0	4	100.0
	Not using	104	78.8	28	21.2	132	100.0
	Test statistic, p-value	Fisher’s exact test P-value = 0.581					

## 4 Discussion

Gender and age were significantly associated with medication adherence (Table 1). Medication adherence among women (85.9%) was higher than among men. It was also higher among patients below 60 years of age than among those 60 years of age or older. The results of this study are consistent with the findings reported by Horri *et al*., who suggested that medication adherence is higher in patients aged less than 60 years [20]. In contrast, Chew *et al*. (2015) concluded that medication adherence increases in patients older than 50 years [21]. This difference may be due to the larger number of patients aged above 50 years (492 people) compated to those aged 50 years or younger (174 people).

There was a significant difference in medication adherence between patients with different income levels (p = 0.016). Medication adherence was higher in patients with higher income levels. Similarly, Shams *et al*. found a significant relationship between income level and medication adherence (p < 0.01), and adherence was also higher among people with higher income [22]. In contrast, Aloudah *et al*. reported no significant association between income and adherence to hypoglycemic drugs [15]. This inconsistency may exist because the variation in the subjects’ income was minor in the study by Aloudah *et al*.

Furthermore, the results suggest that there is a significant difference in medication adherence between the patients with a hemoglobin A1c level of less than 7% and those with a level greater than or equal to 7% (p = 0.010). Adherence was higher in patients with a hemoglobin A1c level of less than 7%. Gorden *et al*. also found a significant association of medication adherence with hemoglobin A1c. In patients reporting medication adherence, hemoglobin A1c level was lower and glycemic control was better [16]. The results of this study are consistent with the findings reported by Horri *et al*. and Aoudah *et al*. [15, 20].

The results also suggest a significant association of medication adherence with patients without hypoglycemia (p = 0.0001). This finding may be explained by the fact that most of these people used glibenclamide, a member of the class of sulfonylureas, which is one of drugs most frequently associated with hypoglycemia. Fadheel *et al*. reported that hypoglycemia is more prevalent in patients with low medication adherence [23]. This finding is consistent with the results of the present study. Gorden *et al*. concluded that hypoglycemia is more probable in patients reporting medication adherence [16]. This finding is inconsistent with the results of our study.

Among the 136 patients in the present study, 50 (36.8%) reported a medication period of 10 years or more, and 86 (63.2%) reported a medication period of less than 10 years. According to the results of the chi-square test, there was a significant difference in medication adherence between patients with different medication periods (p = 0.041); adherence was higher among patients who had been taking the drugs for less than 10 years. The results of the present study are consistent with the findings of Boccuzzi *et al*. in America [24] as well as Jemal *et al*. [25] and Ayele *et al*. [26] in Ethiopia.

**Figure 1.**
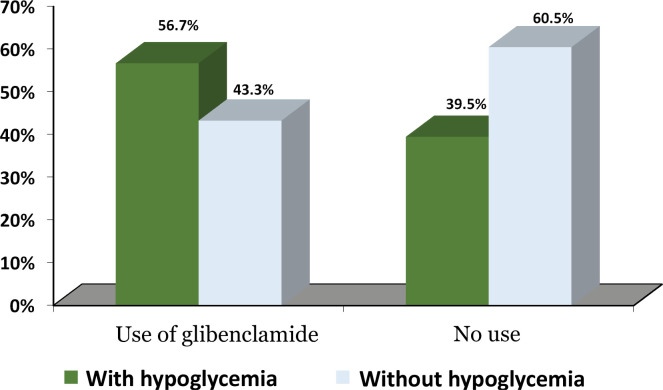
The figure shows the relationship between the use of glibenclamide and hypoglycemia. Hypoglycemia was significantly higher in patients using glibenclamide (p = 0.048).

As shown by the results, metformin was taken by 111 people (81.6%), glibenclamide by 60 (44.1%), gliclazide by 25 (18.4%), sitagliptin by 15 (11.0%), repaglinide by 7 (5.1%), pioglitazone by 4 (2.9%), and linagliptin by 3 (2.2%). According to the results (Figure 1), there was a significant difference in medication adherence between patients using and those not using glibenclamide (p = 0.048); adherence was reported to be lower in patients using glibenclamide. This finding can be associated with the fact that glibenclamide may have caused hypoglycemia and that the patients have therefore not used the drug according to the instructions. Gorden *et al*. found that with dipeptidyl peptidase-4 inhibitor, medication adherence was higher than with other single drugs [16]. This finding is inconsistent with the results of our study. Further studies should be conducted to investigate this issue in more detail.

## 5 Conclusions

According to the findings of the present study, there was no significant relationship between medication adherence and using concomitant drugs, FBG, and the number of hypoglycemic tablets used daily. However, a higher rate of medication adherence was reported in women than in men and in patients aged less than 60 years old than in those aged 60 years or older. Furthermore, adherence was higher among patients with higher incomes. Meanwhile, a higher level of adherence was reported in patients with a hemoglobin A1c level of less than 7%, a medication period of less than 10 years, and those reporting no hypoglycemia. A comparison of the type of drugs suggested that patients using glibenclamide report a lower adherence, which may be due to the fact that these drugs tend to cause hypoglycemia.

## References

[1] Chu WM, Ho HE, Huang KH, Tsan YT, Liou YS, Wang YH, Lee MC, Li YC. The prescribing trend of oral antidiabetic agents for type 2 diabetes in Taiwan: An 8-year population-based study. Medicine (Baltimore) 2017 96(43):e8257. 29068991 10.1097/MD.0000000000008257 PMC5671824

[2] Kennedy-Martin T, Boye KS, Peng X. Cost of medication adherence and persistence in type 2 diabetes mellitus: a literature review. Patient Prefer Adherence 2017. 11:1103. 28721024 10.2147/PPA.S136639 PMC5501621

[3] Jeannette AC, Esther J, Anoop MT, et al. IDF Diabetes Atlas, 8th ed., International Diabetes Federation, Brussels, 2017.

[4] World Health Organization. Diabetes fact sheet. Available from: http://www.who.int/mediacentre/factsheets/fs312/en/. Accessed May 11, 2017.

[5] De Brito GM, Gois CF, Zanetti ML, Resende GG, Silva JR. Quality of life, knowledge and attitude after educational program for diabetes. Acta Paulista de Enfermagem 2016. 29(3):298-306.

[6] de Araujo MF, de Jesus dos Santos Alves P, Veras VS, de Araujo TM, Zanetti ML, Damasceno MM. Drug interactions in Brazilian type 2 diabetes patients. Int J Nurs Pract 2013. 19(4):423-30. 23915412 10.1111/ijn.12078

[7] Babazadeh T, Dianatinasab M, Daemi A, Nikbakht HA, Moradi F, Ghaffari-fam S. Association of self-care behaviors and quality of life among patients with type 2 diabetes mellitus: Chaldoran County, Iran. Diabetes Metab J 2017. 41(6):449-456. 29272083 10.4093/dmj.2017.41.6.449 PMC5741554

[8] Dawed AY, Zhou K, Pearson ER. Pharmacogenetics in type 2 diabetes: influence on response to oral hypoglycemic agents. Pharmgenomics Pers Med 2016. 9:17-29. 27103840 10.2147/PGPM.S84854 PMC4827904

[9] Awodele O, Osuolale JA. Medication adherence in type 2 diabetes patients: study of patients in Alimosho General Hospital, Igando, Lagos, Nigeria. Afr Health Sci 2015. 15(2):513-522. 26124798 10.4314/ahs.v15i2.26 PMC4480454

[10] Nau DP. Recommendations for improving adherence to type 2 diabetes mellitus therapy-focus on optimizing oral and non-insulin therapies. Am J Managed Care 2012. 18(3 Suppl):S49-S54. 22558942

[11] Bailey C, Kodack M. Patient adherence to medication requirements for therapy of type 2 diabetes. Int J Clin Pract 2011. 65(3):314-322. 21314869 10.1111/j.1742-1241.2010.02544.x

[12] Krass I, Schieback P, Dhippayom T. Adherence to diabetes medication: a systematic review. Diabetic Med 2015. 32(6):725-737. 25440507 10.1111/dme.12651

[13] Giorgino F, Penfornis A, Pechtner V, Gentilella R, Corcos A. Adherence to antihyperglycemic medications and glucagon-like peptide 1-receptor agonists in type 2 diabetes: clinical consequences and strategies for improvement. Patient Prefer Adherence 2018. 12:707. 29765207 10.2147/PPA.S151736 PMC5944456

[14] Asif M. The prevention and control the type-2 diabetes by changing lifestyle and dietary pattern. J Educ Health Promot 2014. 3:1. 24741641 10.4103/2277-9531.127541 PMC3977406

[15] Aloudah NM, Scott NW, Aljadhey HS, Araujo-Soares V, Alrubeaan KA, Watson MC. Medication adherence among patients with Type 2 diabetes: A mixed methods study. PLoS One 2018. 13(12):e0207583. 30533042 10.1371/journal.pone.0207583 PMC6289442

[16] Gordon J, McEwan P, Idris I, Evans M, Puelles J. Treatment choice, medication adherence and glycemic efficacy in people with type 2 diabetes: a UK clinical practice database study. BMJ Open Diabetes Res Care 2018. 6(1):e000512. 10.1136/bmjdrc-2018-000512 PMC5942418 29755756

[17] Association AD. 8. Pharmacologic approaches to glycemic treatment: Standards of Medical Care in Diabetes 2018. Diabetes Care 2018. 41(Suppl 1):S73. 29222379 10.2337/dc18-S008

[18] Cersosimo E, Johnson EL, Chovanes C, Skolnik N. Initiating therapy in patients newly diagnosed with type 2 diabetes: Combination therapy vs a stepwise approach. Diabetes Obes Metab 2018. 20(3):497-507. 28862799 10.1111/dom.13108

[19] Fadare J, Olamoyegun M, Gbadegesin B. Medication adherence and direct treatment cost among diabetes patients attending a tertiary healthcare facility in Ogbomosho, Nigeria. Malawi Med J 2015. 27(2):65-70. 26405515 10.4314/mmj.v27i2.7 PMC4562083

[20] Horii T, Momo K, Yasu T, Kabeya Y, Atsuda K. Determination of factors affecting medication adherence in type 2 diabetes mellitus patients using a nationwide claim-based database in Japan. PloS One 2019. 14(10): e0223431. 31593574 10.1371/journal.pone.0223431 PMC6782087

[21] Chew BH, Hassan NH, Sherina MS. Determinants of medication adherence among adults with type 2 diabetes mellitus in three Malaysian public health clinics: a cross-sectional study. Patient Pref Adherence 2015. 9:639. 10.2147/PPA.S81612 PMC4427255 25999699

[22] Shams ME, Barakat EA. Measuring the rate of therapeutic adherence among outpatients with T2DM in Egypt. Saudi Pharmaceut J 2010. 18(4):225-232. 10.1016/j.jsps.2010.07.004 PMC3730985 23960731

[23] Fadheel J, Mohammed AJ. Adherence to therapy among Iraqi patients with type 2 DiabetesMellitus. J Health Med Nurs 2016. 32:75-82.

[24] Boccuzzi SJ, Wogen J, Fox J, Sung JC, Shah AB, Kim J. Utilization of oral hypoglycemic agents in a drug-insured US population. Diabetes Care 2001. 24(8):1411-1415. 11473078 10.2337/diacare.24.8.1411

[25] Jemal A, Abdela J, Sisay M. Adherence to oral antidiabetic medications among type 2 diabetic (T2DM) patients in chronic ambulatory wards of Hiwot Fana Specialized University Hospital, Harar, Eastern Ethiopia: a cross sectional study. J Diabetes Metab 2017. 8:721.

[26] Ayele AA, Tegegn HG, Ayele TA, Ayalew MB. Medication regimen complexity and its impact on medication adherence and glycemic control among patients with type 2 diabetes mellitus in an Ethiopian general hospital. BMJ Open Diabetes Res Care 2019. 7(1):e000685. 10.1136/bmjdrc-2019-000685 PMC6606061 31321061

